# A novel dataset and efficient deep learning framework for automated grading of renal cell carcinoma from kidney histopathology images

**DOI:** 10.1038/s41598-023-31275-7

**Published:** 2023-04-07

**Authors:** Amit Kumar Chanchal, Shyam Lal, Ranjeet Kumar, Jin Tae Kwak, Jyoti Kini

**Affiliations:** 1 Department of Electronics and Communication Engineering, National Institute of Technology Karnataka, Surathkal, Mangaluru, Karnataka 575025 India; 2 School of Electronics Engineering, Vellore Institute of Technology, Chennai, Tamil Nadu India; 3 School of Electrical Engineering, Korea University, Seoul, Korea; 4 Department of Pathology, Kasturba Medical College, Mangalore, India; 5 Manipal Academy of Higher Education, Manipal, India

**Keywords:** Cancer, Health care, Medical research, Oncology

## Abstract

Trends of kidney cancer cases worldwide are expected to increase persistently and this inspires the modification of the traditional diagnosis system to respond to future challenges. Renal Cell Carcinoma (RCC) is the most common kidney cancer and responsible for 80–85% of all renal tumors. This study proposed a robust and computationally efficient fully automated Renal Cell Carcinoma Grading Network (RCCGNet) from kidney histopathology images. The proposed RCCGNet contains a shared channel residual (SCR) block which allows the network to learn feature maps associated with different versions of the input with two parallel paths. The SCR block shares the information between two different layers and operates the shared data separately by providing beneficial supplements to each other. As a part of this study, we also introduced a new dataset for the grading of RCC with five different grades. We obtained 722 Hematoxylin & Eosin (H &E) stained slides of different patients and associated grades from the Department of Pathology, Kasturba Medical College (KMC), Mangalore, India. We performed comparable experiments which include deep learning models trained from scratch as well as transfer learning techniques using pre-trained weights of the ImageNet. To show the proposed model is generalized and independent of the dataset, we experimented with one additional well-established data called BreakHis dataset for eight class-classification. The experimental result shows that proposed RCCGNet is superior in comparison with the eight most recent classification methods on the proposed dataset as well as BreakHis dataset in terms of prediction accuracy and computational complexity.

## Introduction

Kidney cancer is currently considered to be the leading cause of cancer and Renal Cell Carcinoma (RCC) is the most common among all kidney cancer cases. Statistics and estimate^1,2^ indicate increasing new cases of kidney cancer worldwide and therefore there is an essential need of a fast and precise cancer detection system to deal the future challenges. Figure out the stage and grade of the renal tumor is an important prognostic parameter for the diagnosis of kidney cancer. Identification of the stage is more about the tumor size, location, and how far cancer has been spread to the nearby lymph nodes, whereas the cancer grade describes how different or abnormal the cancerous cells look compared to normal healthy cells under the microscope. With the proper knowledge of stage and grade, the pathologists get an idea that how quickly it will grow and how much it will spread to the other parts of the body, and the doctor can plan the treatment accordingly. The manual grading of complex histologic patterns of surgical slides is a tedious task and it is prone to contradictions and errors hence it requires highly specialized pathologists. A fully automated and precise method of grading of kidney cancer from histopathology images is in high demand for identifying malignant tumors. According to Fohrman’s 4-tier grading system used by Hong et al.^3^, the Grade-1 nuclei are of tubuler structure and uniform and it is very similar to normal nuclei. Grade-2 nuclei have slightly irregular contour compared to normal nuclei. Grade-3 nuclei have more irregular contour. Grade-4 nuclei have pleomorphic cells, mitoses, multilobate with the Grade-3 feature. Another method of grading system by WHO/international society of urologic pathology (ISUP)^4^, where Grade-1 to Grade-3 tumor is decided based on nuclear prominence whereas Grade-4 is recognized by the presence of those cells showing extreme nuclear pleomorphism. The ISUP nucleolar grading system has been shown superior to the traditional Fuhrman grading system by Delahunt et al.^5^ and recommended as the main basis for grading by pathologists. Visualization of nuclear morphology, nucleolar prominence, and nuclear membrane irregularities of different grades of renal tumor is presented in Table 1.

**Table 1:** Visualization of different grades of renal tumor (normal to Grade-4 from left to right).

Normal (Grade-0)	Grade-1	Grade-2	Grade-3	Grade-4
				
The cells of the proximal tubules have central nuclei and very acidophilic cytoplasm	Nucleoli are basoph	Nucleoli are seen as eosinophilic at 400$\times$ magnification but not very prominant at 100$\times$ magnification	Nucleoli conspicuous and eosinophilic at 100$\times$ magnification	Pronounced nuclear pleomorphism
Cells are well arranged and are normal in number	Nucleoli are not visible even 400$\times$ magnification Nucleoli are seen as eosinophilic at 400$\times$ magnification but not very prominant at 100$\times$ magnification	Slightly irregular contour compared to normal nuclei	Clearly visible tumors were graded as grade-3	Rhabdoid or sarcomatoid differentiation
A normal glomerulus structure	Morphology is very similar to normal nuclei	Grade-3 nuclei have a more irregular contour compared to normal nuclei		Contains tumor giant cells

The application of deep learning to analyze the histopathological images of kidney, breast, liver, prostate, colon, and other organs include a number of tasks such as nuclei detection and segmentation, characterization of subtypes of cancer, and grading. A few recent works applied convolutional neural networks and emphasis on kidney cancer and grading. For example, RCC classification^6^ exploits pre-trained ResNet-34 and directed acyclic graph classifier for three subtypes of RCC on TCGA data. Another common method^7^ adopts ResNet-18 for the classification of five related subtypes of RCC. The method^8^, designed a lasso regression-based classification model to differentiate the associated grades of clear cell RCC. The works^9–11^ utilize the transfer learning techniques for the analysis of breast cancer histopathology images and transfers ImageNet weight on a deep learning model like ResNet50^12^, DenseNet121^13^, Inceptionv3^14^, and Inception ResNetv2^15^. A three-layer convolutional neural network^16^ is designed to detect invasive tumors on breast histopathology data. For binary classification of breast cancer, DBN^17^ used principal component analysis for the extracted features through an unsupervised pre-training. The works BHC^18^ and BreastNet^19^ employ efficient CNN modules namely small SE ResNet, CBAM, attention module, and residual block for multi-class classification of BreaKHis data. These efforts have inspired the further design of end-to-end trainable deep learning networks. LiverNet^20^ employs atrous spatial pyramid pooling block in addition to attention and residual module used in previous work. The work by Hameed et al.^21^ leverages the strength of inception and residual connections, and the applied method is computationally efficient using depth-wise separable convolution. The concept of attention in deep learning has been widely explored in recent decades. To focus on the most informative component of the input images, the attention or gating mechanism is incorporated in diverse application domains. CBAM^22^ composed of cascaded channel attention and spatial attention module that extended the idea of attention in both dimensions. The average pooled and max pooled features are utilized to produce activation map in CBAM. In two steps called squeeze and excitation, SENet^23^ scaled the globally pooled information with the given input to highlight the channel activation map. The proposed method utilizes global information of each channel in a very simple manner for giving attention to the most relevant morphological features of RCC. Recently, the shuffling of channels within a layer was exploited for efficient model design in convolutional neural network. The shuffle operation proposed^24,25^ is a stack of channel shuffle units and group convolution, and works better under smaller computational budgets. To meet different target complexities, these techniques effectively utilize many convolution groups and the advantages of adjustment of channels. Some works^26,27^ adopts shuffle unit and applied various attention mechanism to the shuffled version of multiple sub-features. The idea is effective since it enables sufficient communication across channels and helps in object detection by information communication between sub-features. The use of lightweight transformers, self-attention mechanisms, and ensemble learning strategies are the innovative ideas reported in^28–30^ which show effectiveness on various medical image modalities. The effect of transfer learning and vision transformer^31^ is also studied and comparable experiments are performed. Inspired by the above-related work, this study proposes an end-to-end trained deep learning model for grading of clear cell RCC from kidney histopathology images.

Most of the previous work focused on transfer learning techniques and using pre-trained weights of the ImageNet dataset. There is no publicly available dataset for grading of clear cell RCC. Pre-trained ImageNet weights transfer powerful texture features, in spite of this for improved classification accuracy, it is required to give more attention to its nucleolar morphology and prominence. The proposed method is intended to show how deep learning can be applied effectively to differentiate kidney histopathology images into five categories namely Normal/Non-cancerous (Grade-0), Grade-1, Grade-2, Grade-3, and Grade-4. Compared with previous work, our approach stands out due to the following reasons (1) instead of shuffling, the proposed method shares the information between different layers. (2) A part of the previous layer feature map is used as a skip connection to the next higher-order layer. (3) Sharing of inter-channel information with two different paths can be viewed as a beneficial supplement to each other and it produces rich local variations. (4) The proposed CNN block can be combined with other deep learning techniques to further advance the performance. The proposed work addresses the limitations of previous methods. Our contribution in this study is as follows:

1. A most accurate and efficient end-to-end fully automated deep learning architecture is proposed for grading renal tumors from H &E stained kidney histopathology images.
2. This study proposes a novel CNN block called shared channel residual (SCR) block which shares the information between different layers and strengthens the local semantic features at multiple stages within the network. This block contributes maximally to the proposed method.
3. A new benchmark RCC dataset of H &E stained kidney histopathology images is created for grading of renal tumors, obtained from the Department of Pathology, KMC Mangalore, Manipal Academy of Higher Education (MAHE), Manipal, Karnataka, India.
4. Comparable experiments performed on multiple organ histopathology datasets include transfer learning methods, deep learning networks trained from scratch, and Vision Transformer (ViT) method. The proposed RCCGNet requires reduced computational resources and outperforms the eight most recent benchmark models in terms of prediction accuracy.

**Figure 1:**
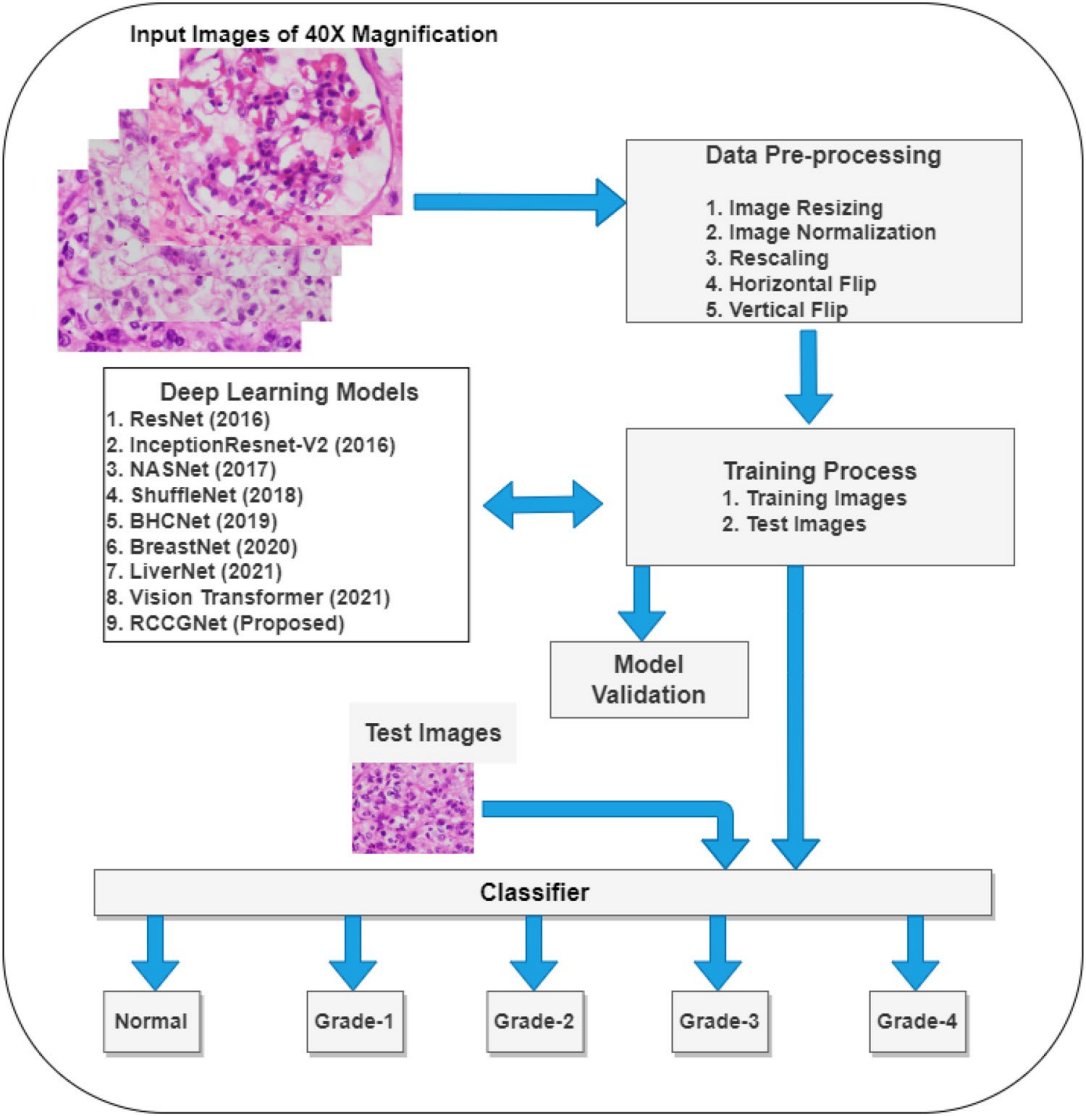
Grading pipeline of proposed RCCGNet.

The methodology of the proposed work has been pipelined as shown in Fig. 1. The workflow of this paper is as follows: “Methods” section describes the proposed novel dataset, training, implementations along with the proposed model. “Results” section compares the results of the proposed RCCGNet with other state-of-the-art classification techniques, and “Discussion” section includes the impact of important components of the proposed network through an ablation study, statistical analysis, and computational complexity analysis.

## Methods

### Dataset and image processing

The introduced KMC kidney histopathology dataset includes non-cancerous (Grade-0) and cancerous (Grade-1 to Grade-4) images of the Renal Clear Cell Carcinoma. These images were collected from October 2020 to December 2022 as a part of a clinical study at Department of Pathology, Kasturba Medical College (KMC) Mangalore, Manipal Academy of Higher Education (MAHE), Manipal, Karnataka, India. This project was approved by the institutional ethics committee Kasturba Medical College (KMC), Mangalore, protocol no-IEC KMC MLR 02/2022/57. The conducted research reported in this article involving human participants was in accordance with the ethical standards of the institutional and/or national research committee and with the 1964 Helsinki declaration and its later amendments or comparable ethical standards. Regarding data, informed consent from all the patients was obtained for conducting the experiments, and the personal details were protected.

The samples were collected by surgical (open) biopsy (SOB) of kidney tissue, stained with hematoxylin and eosin (H &E). These histopathology slides are classified into Normal/Non-cancerous (Grade-0), Grade-1, Grade-2, Grade-3, Grade-4 and labeled through a careful clinical study in the department of pathology in KMC. The procedure used by the pathologists here is the most commonly used paraffin procedure. The complete preparation procedure includes steps such as fixation, dehydration, clearing, infiltration, embedding, and trimming^32^. After the preparation process, the pathologist visually identified the tumoral areas in each slide under a microscope. An olympus BX-50 system microscope with a relay lens and a magnification of 3.3$\times$ coupled to a olympus digital color camera DP-22 is used to obtain digitized images from the kidney tissue slides. This camera uses a 1/1.8” colour CCD (Charge-Coupled Device) with pixel size 3.69 $\times$ 3.69 and a total pixel number of 1920 $\times$ 1440 $\times$ 3. This dataset has been annotated by a group of pathologists at KMC, Manipal. Pre-setting was kept automatic and except for the selection of area, everything was the same for all images. Non-overlapping square patches were extracted and resized to a size of 224 $\times$ 224 and normalized to zero mean and unit variance before passing them through deep learning architecture for training and testing. The entire dataset is splitted into training and test sets. Approximately 80% of the total images were considered for training, while the rest 20% for the test. The validation set was created by cropping non-overlapping regions of training patches. The data augmentation techniques, horizontal and vertical flipping were used on training set to further increase the diversity. After random cropping and data augmentation, 3442 patches belonging to five classes were obtained as training set. Out of these, 693 patches are of Normal/Non-cancerous grade, 708 patches belong to grade-1 cancer, 648 patches belong to grade-2 cancer, 735 patches belong to grade-3 and, 648 patches belong to grade-4 kidney cancer. Data augmentation on the test set has not been applied and model evaluation performed on an original test set. The details of the grade distribution of the proposed data are presented in Table 2. To verify the validity of the proposed RCCGNet on the proposed novel dataset, we also analyzed the performance of the RCCGNet on the BreaKHis dataset. BreaKHis dataset^19^ is a well-established publicly available breast cancer histopathology dataset used in various state-of-the-art deep learning models.

**Table 2:** Proposed dataset grades distribution.

Type	Training patches	Test patches
Grade0	162	41
Grade1	108	27
Grade2	99	25
Grade3	96	24
Grade4	112	28
Total	577	145

### Training and implementation

The proposed RCCGNet and all comparison model were implemented on a Dell-G4G3NSM workstation with 8 GB NVIDIA QUADRO P4000 GPU and 64 GB RAM. All of our training processes performed on Python-3, TensorFlow 2.4.0, Keras an open source platform. Experimentation of our RCCGNet consists of three stages: training, testing and validation. For a given range of hyperparameters, the network was trained and measured the performance based on the test set.

**Table 3:** Details of hyper parameters.

Optimizer	Adam with initial learning rate of 0.001
Batch size	4
Convolution filter	16, 32, 64
Total epochs	65
Reduce learning rate	Factor = 0.5, patience = 5, min-delta = 0.0001, monitor-validation acc.
Early stop	Patience = 30, min-delta = 0.0001, monitor-validation acc., restore-best-weights

The proposed RCCGNet model is more efficient with the Adam optimizer, with the default learning rate of 0.001. Three important parameters namely reduced learning rate, model checkpoint and early stopping were used effective training. Reduced learning rate helps to schedule the learning rate with the progress of training. We can choose a measure that we would like to monitor like validation accuracy, validation loss, etc. For our purpose, we monitored validation accuracy, and if it is not improving in 5 successive epochs with a minimum change of 0.0001, this callback reduces the current learning rate. The new learning rate will be half of the previous learning rate and this process continues till the last epoch. Another callback is model checkpoint, which helps in saving the model weight, which is the best fit for our data. Validation accuracy is continuously monitored and the model checkpoint saves the best weight on the provided file path. The early stopping method helped to decide the total number of epochs that we trained the model for given data. Less training probably leads to underfitting, while excessive training may cause overfitting. After 65 epochs there is no improvement in performance has been observed for all reference models. In this way, all reference models and proposed model were trained for 65 epochs. Another hyper parameter is batch size, larger batch size allows to parallelize computations to a greater degree but lead to poor generalization. The proposed model giving satisfactory results for batch size 4. The important controlling hyperparameter that we tuned is shown in Table 3. The python implementation code is available at https://github.com/shyamfec/RCCGNet.

### Loss function and evaluation metrics

The process of optimizing loss function quantifies the error between the output of the network and the corresponding labels. Cross-entropy loss followed by sigmoid activation is called sigmoid cross-entropy loss which is used for two-class classification problems. Similarly, cross-entropy loss followed by softmax activation is called softmax cross-entropy that is useful for multiclass classification problems. Here, softmax cross-entropy loss function is used for the classification of kidney histopathology images into five categories namely Normal/Non-cancerous (Grade-0), Grade-1, Grade-2, Grade3, and Grade-4. Equation (1) is a standard cross-entropy formula used in^33^, where $y_{i}$ is target value and $f(x)_{i}$ indicates output of activation function equivalent to probability of each class.

1 $$\begin{aligned} Cross\,Entropy\,Loss = -\sum _{i=1}^{C}y_{i}log(f(x)_{i}) \end{aligned}$$

For an input vector $x_{i}$ with length equal to the number of classes c, $x=(x_{1},x_{1},\ldots x_{c})\in {\mathbb {R}}^{c}$, the output function of the softmax $w_{\phi }(x_{i})$ can be expressed in Eq. (2) and here the $c=5$ to divide the probability map into five classes.

2 $$\begin{aligned} w_{\phi }(x_{i})=\begin{bmatrix} p(y_{i}=1)\, |x_{i},\phi \\ p(y_{i}=2)|x_{i},\phi \\ \vdots \\ p(y_{i}=c)|x_{i},\phi \end{bmatrix}=\frac{1}{\sum _{j=1}^{c}e^{\phi _{j}^{T}x_{i}}}\begin{bmatrix} e^{\phi _{1}^{T}x_{i}} \\ e^{\phi _{2}^{T}x_{i}} \\ \vdots \\ e^{\phi _{c}^{T}x_{i}} \end{bmatrix} \end{aligned}$$

The term $\frac{1}{\sum _{j=1}^{c}e^{\phi _{j}^{T}x_{i}}}$ is used to normalized the output to 1 and $\Phi$ is the parameter of softmax classifier^34^.

For evaluating the model, four different performance metrics namely *Accuracy*, *Precision*, *Recall*, and $F_{1},(\beta =1)$^35,36^ were used. The model predictions are finally grouped into four, namely True Positive (TP), False Positive (FP), True Negative (TN), and False Negative (FN). The performance metrics can be expressed in terms of these four groups as in Eqs. (3), (4), and (5).

3 $$\begin{aligned} Acc&=\frac{TP_{count}+TN_{count}}{TP_{count}+TN_{count}+FP_{count}+FN_{count}} \end{aligned}$$

4 $$\begin{aligned} Pre&=\frac{TP_{count}}{TP_{count}+FP_{count}}, Re=\frac{TP_{count}}{TP_{count}+FN_{count}} \end{aligned}$$

5 $$\begin{aligned} F_{1}\, score&=\frac{2.TP_{count}}{2.TP_{count}+FP_{count}+FN_{count}} \end{aligned}$$

**Figure 2:**
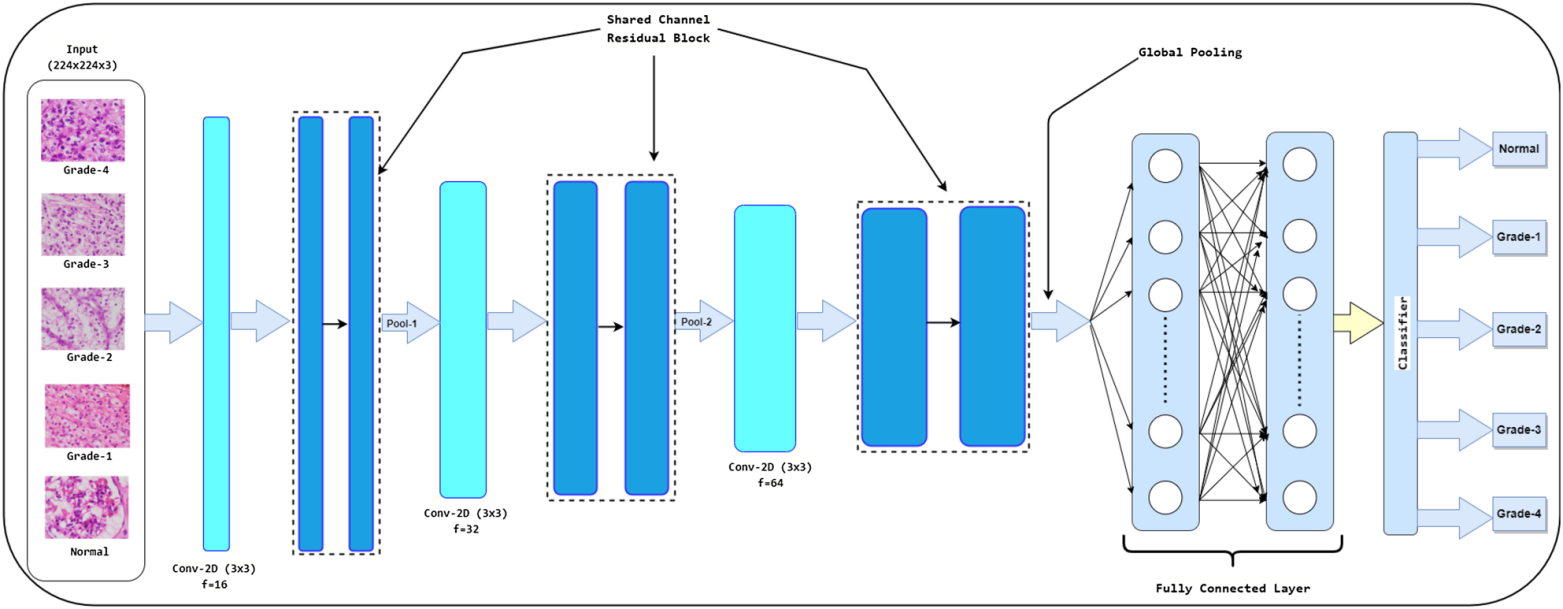
Structure of the proposed RCCGNet for grading of renal cell carcinoma from kidney histopathology images.

### Proposed architecture

Recently, deep learning and artificial intelligence-based system achieved new heights with the development of a highly optimized algorithm which leads to encouraging results in the field of digital pathology. The proposed Renal Cell Carcinoma Grading Network (RCCGNet) comprises of three foremost stages that are (1) data preparation; (2) shared channel residual (SCR) block; (3) finally, grading phase where the network differentiates the five different grades of renal tumors by using a softmax non-linear activation function. The proposed model is designed by integrating SCR block, Conv2D, and fully connected layers to extract the features from the kidney histopathology images. The structure of the proposed RCCGNet for grading of RCC from kidney histopathology images has been shown in Fig. 2.

### Shared channel residual block

This section describes the designed architecture of the proposed shared channel residual (SCR) block. The SCR block employed in the proposed RCCGNet contains convolutional layers, a new and efficient method of sharing the information between two different layers, a simple gating mechanism to focus on the most relevant morphological features of RCC, and a residual connection. The first stage of the SCR block accepts an input, which undergoes a convolution operation. After that, both the input and the feature map obtained after the convolution operation are divided into two groups channel-wise. In the next step of operation, group-1 of the lower layer information is concatenated with group-2 of the higher layer feature map. Similarly, group-1 of higher layer information is also concatenated with group-2 of lower layer data. In this way, the SCR block with two concatenated layers forms two parallel paths and gives the advantage of skip connection with the enhanced features by sharing the channel between two layers. The proposed SCR network is different from previous work^24–27^ which shuffles the channel within a layer, whereas the SCR block shares the information between two different layers. The resultant feature of the SCR block is the combination of lower-order features and higher-order features. Further, the SCR block allows to learn feature maps associated with different versions of the input with two parallel paths and one skip connection. Following are the advantages of the proposed SCR block over the other related existing methods. (1) The proposed method is scalable since the feature map can be split into any number of groups and also it can be shared with any higher-layer feature map. The architecture, in this study, is designed especially for grading of RCC histopathology application. (2) SCR block can be incorporated into any other deep learning architecture to improve accuracy. (3) Information sharing between different layers, a part of the previous layer data is used as a skip connection which strengthens the local semantic features at different stages of the network. (4) There is no additional computational complexity involved in sharing the information between two different layers.

**Figure 3:**
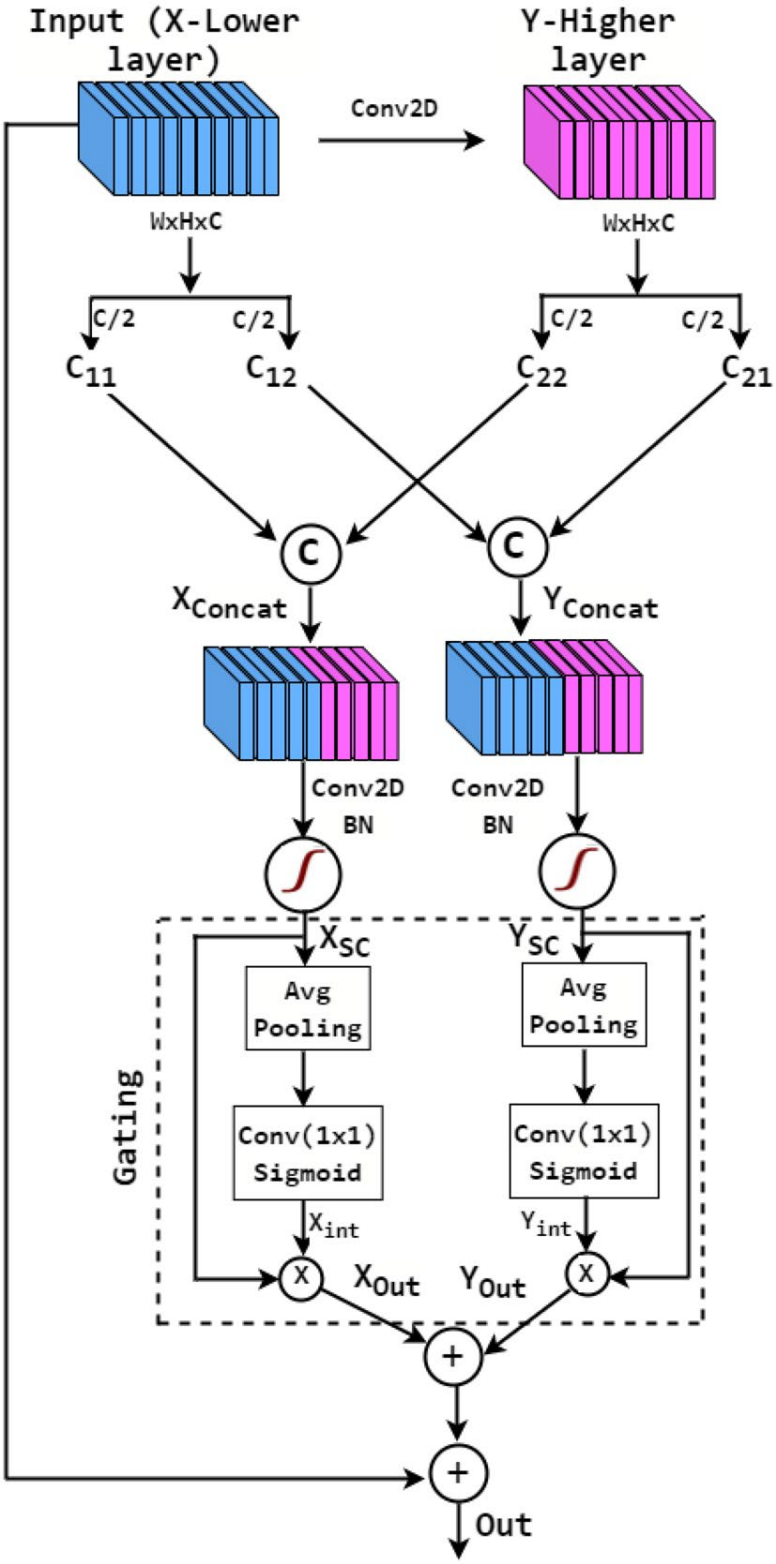
Shared channel residual block.

### Mathematical representation of the proposed SCR block

In Fig. 3, the input feature map *X* is transformed to *Y* by convolution operation, which is expressed in Eq. (6), where $F_{tr(3 \times 3)}$ is a convolutional operator of kernel size (3 $\times$ 3).

6 $$\begin{aligned} X\in \mathbb {R^{H\times W\times C}} \mathop {\xrightarrow {}}^{\mathrm {F_{tr(3 \times 3)}}}_ Y\in \mathbb {R^{H\times W\times C}} \end{aligned}$$

The kernel $K=\left[ k_{1},k_{2}, k_{3}\ldots k_{C} \right]$ is an optimizable feature extractor, applied at each channel and each image position. $k_{c}$ refers to the *C*th kernel of *K*. Here *C* = 16, 32, and 64 channels are used at three different stages of the network. *Y* is the result of convolution operation ($*$) of input feature map *X* with kernel *K* represented in Eq. (7).

7 $$\begin{aligned} Y=\left[ y_{1}, y_{2}\ldots y_{C} \right] =X*K \end{aligned}$$

Equation (8) represents convolution of a single feature map in *X*.

8 $$\begin{aligned} y_{c}=k_{c}*X=\sum _{s=1}^{C}k_{c}^{s}*x^{s} \end{aligned}$$

where $k^{c}=\left[ k_{c}^{1},k_{c}^{2}\ldots k_{c}^{C} \right]$, $X=\left[ x^{1},x^{2}, x^{3}\ldots x^{C} \right]$, $y_{c}\in \mathbb {R^{H\times W}}$, and $k_{c}^{s}$ is a single channel of $k_{c}$.

Now *X* and *Y* are divided into $C_{11}, C_{12}$, $C_{21}, C_{22}$ channel-wise, where $C_{11}, C_{12}, C_{21}, C_{22}\in {\mathbb {R}}^{\mathbb {H\times W\times C}/{2}}$ are shown below in Eqs. (9) to (12).

9 $$\begin{aligned} C_{11}= & {} \left[ x^{1},x^{2},x^{3}\ldots x^{\frac{C}{2}} \right] \end{aligned}$$

10 $$\begin{aligned} C_{12}= & {} \left[ x^{\frac{C}{2}+1},x^{\frac{C}{2}+2},x^{\frac{C}{2}+3}\ldots x^{C} \right] \end{aligned}$$

11 $$\begin{aligned} C_{21}= & {} \left[ y^{1},y^{2},y^{3}\ldots y^{\frac{C}{2}} \right] \end{aligned}$$

12 $$\begin{aligned} C_{22}= & {} \left[ y^{\frac{C}{2}+1},y^{\frac{C}{2}+2},y^{\frac{C}{2}+3}\ldots y^{C} \right] \end{aligned}$$

The lower order features and deeper layer features are shared and concatenated at three different stages of the network and represented using the below-mentioned Eqs. (13), and (14), where $\copyright$ represents concatenation operation
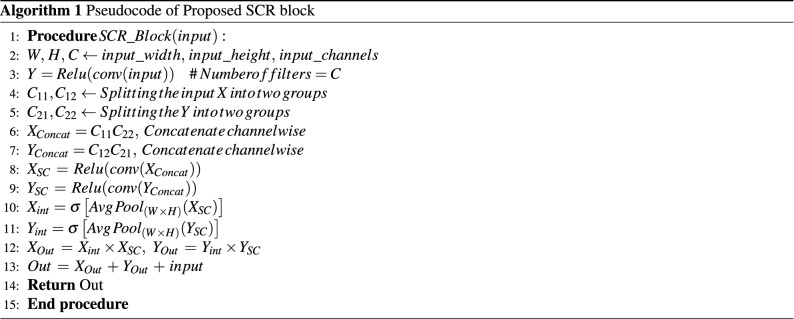
.

13 $$\begin{aligned} X_{Concat}= & {} C_{11}\copyright C_{22}= \left[ x^{1},x^{2}\ldots x^{\frac{C}{2}}, y^{\frac{C}{2}+1}\ldots y^{C} \right] \end{aligned}$$

14 $$\begin{aligned} Y_{Concat}= & {} C_{11}\copyright C_{22}= \left[ y^{1},y^{2}\ldots y^{\frac{C}{2}}, x^{\frac{C}{2}+1}\ldots x^{C} \right] \end{aligned}$$

The concatenated features are fed to a sequence of operations called Conv2D, Batch Normalization (BN), and Rectified Linear Unit (ReLU) and presented in Eqs. (15), (16).

15 $$\begin{aligned} X_{SC}= & {} ReLU\left[ F_{tr(3\times 3)} \left( X_{Concat} \right) \right] \end{aligned}$$

16 $$\begin{aligned} Y_{SC}= & {} ReLU\left[ F_{tr(3\times 3)} \left( Y_{Concat} \right) \right] \end{aligned}$$

A kind of gating mechanism is also deployed effectively and uniquely in the SCR block to focus on the most relevant morphological features of RCC and enable contextual information for the network while producing predictions. $X_{SC}$ is given as input to average pooling where the kernel used is $(H\times W)$, so that it is possible to extract the global information of each channel. The resulting feature is convoluted point-wise followed by sigmoid activation. The sigmoid activation assigns scores to each channel according to the relevance of global content associated with the channels. This way, it is giving attention to highly variable nuclear features present in different grades of kidney histopathology images. The extracted global feature calculated from Eqs. (17) and (18) is multiplied by input gating signal $X_{SC}$ and $Y_{SC}$ respectively, and represented in Eqs. (19) and (20).

17 $$\begin{aligned} X_{int}= & {} \sigma \left[ f_{tr\left( 1\times 1 \right) }\left[ Avg.\, Pool_{H\times W}\left\{ X_{SC} \right\} \right] \right] \in {\mathbb {R}}^{1\times 1\times C} \end{aligned}$$

18 $$\begin{aligned} Y_{int}= & {} \sigma \left[ f_{tr\left( 1\times 1 \right) }\left[ Avg.\, Pool_{H\times W}\left\{ Y_{SC} \right\} \right] \right] \in {\mathbb {R}}^{1\times 1\times C} \end{aligned}$$

19 $$\begin{aligned} X_{out}= & {} X_{int}\times X_{SC} \end{aligned}$$

20 $$\begin{aligned} Y_{out}= & {} X_{int}\times Y_{SC} \end{aligned}$$

The final output of the SCR block expressed in Eq. (21) is obtained by adding original input *X* to the output generated by the gating mechanism.

21 $$\begin{aligned} Out=X_{out}+Y_{out}+Input (X) \end{aligned}$$

## Results

The performance of the proposed RCCGNet is compared with the models that uses transfer learning approach as well as deep learning model trained from scratch. Transfer learning methods applies the pre-trained weights of ImageNet dataset. This study includes examination of most recent models through two different organ histopathology datasets called KMC kidney dataset and BreaKHis dataset. ResNet50 network (2016)^12^; InceptionResNetV2 (2016)^15^; and NASNet (2018)^37^; utilizes transfer learning approach, where all the layers are frozen except the few top layers. A global average pooling layer, a dense layer, and a softmax layer were added in place of the final stage of the above models and used to fine-tune the model. The comparision table also includes models, Shufflenet (2018)^24^, BHCNet (2019)^18^, BreastNet (2020)^19^, and LiverNet (2021)^20^, which are end-to-end trained deep learning networks. The comparison table contains a Vision Transformer (2021)^31^, where an image is interpreted as a sequence of patches and processed by a standard transformer encoder. Models trained from scratch does not have any pre-trained weights and all weights and biases were randomly initialized. Experimental results indicate that transferring ImageNet weights to classify the RCC images is not much beneficial. The data preparation phase, morphology, and histopathologic structure of RCC images are completely different from the ImageNet dataset. It would probably be more advantageous if it is possible to transfer the features of histopathology data. Embedding efficient and lightweight CNN module as in^18–20^ gained satisfactory improvement over transfer learning techniques. Table 4 shows performance of different models for grading kidney tissues from KMC kidney histopathology dataset.

**Table 4:** Performance metrics comparison of proposed model with other competitive model (KMC kidney dataset).

Metrics	Transfer learning approach				End-to-end trained deep learning networks					
	Grade	ResNet50 (2016)	IncResV2 (2016)	NASNet (2018)	ShuffleNet (2018)	BHCNet (2019)	BreastNet (2020)	LiverNet (2021)	ViT (2021)	RCCGNet (proposed)
Precision	0	0.8478	0.8260	0.9523	1	0.9743	0.9210	0.9523	0.9743	0.9756
	1	0.8076	0.8095	0.8571	0.9523	0.8181	0.9230	0.92	0.8260	0.8846
	2	0.5937	0.6	0.7666	0.6451	0.75	0.6666	0.6896	0.8421	0.9047
	3	0.7272	0.75	0.4827	0.6	0.75	0.8333	0.7142	0.5666	0.7241
	4	0.7037	0.6071	0.9259	0.96	0.96	1	1	0.8709	1
	Overall	0.7360	0.7185	0.7969	0.8315	0.8508	0.8688	0.8552	0.8160	0.8978
Recall	0	0.9512	0.9268	0.9756	0.9756	0.9268	0.8536	0.9756	0.9268	0.9756
	1	0.7777	0.6296	0.4444	0.7407	1	0.8888	0.8518	0.7037	0.8518
	2	0.76	0.84	0.92	0.8	0.84	0.96	0.8	0.64	0.76
	3	0.3809	0.4285	0.6666	0.7142	0.5714	0.7142	0.7142	0.8095	1
	4	0.6785	0.6071	0.8928	0.8571	0.8928	0.8571	0.8928	0.9642	0.8928
	Overall	0.7097	0.6864	0.7799	0.8175	0.8462	0.8547	0.8469	0.8088	0.89606
F1 Score	0	0.8965	0.8735	0.9638	0.9876	0.9500	0.8860	0.9638	0.9500	0.9756
	1	0.7924	0.7083	0.5853	0.8333	0.9	0.9056	0.8846	0.76	0.8679
	2	0.6666	0.7	0.8363	0.7142	0.7924	0.7868	0.7407	0.7272	0.8260
	3	0.5	0.5454	0.56	0.6521	0.6486	0.7692	0.7142	0.6666	0.84
	4	0.690	0.6071	0.9090	0.9056	0.9259	0.9230	0.9433	0.9152	0.9433
	Overall	0.7093	0.6869	0.7709	0.8186	0.8434	0.8541	0.8493	0.8038	0.8906
Accuracy	0	0.9366	0.9225	0.9788	0.9929	0.9718	0.9366	0.9788	0.9718	0.9859
	1	0.9225	0.9014	0.8802	0.9436	0.9577	0.9647	0.9577	0.9154	0.9507
	2	0.8661	0.8732	0.9366	0.8873	0.9225	0.9084	0.9014	0.9154	0.9436
	3	0.8873	0.8943	0.8450	0.8873	0.9084	0.9366	0.9154	0.8802	0.9436
	4	0.8802	0.8450	0.9647	0.9647	0.9718	0.9718	0.9788	0.9648	0.9788
	Overall	0.7464	0.7183	0.8028	0.8380	0.8661	0.8591	0.8662	0.8239	0.9014

**Figure 4:**
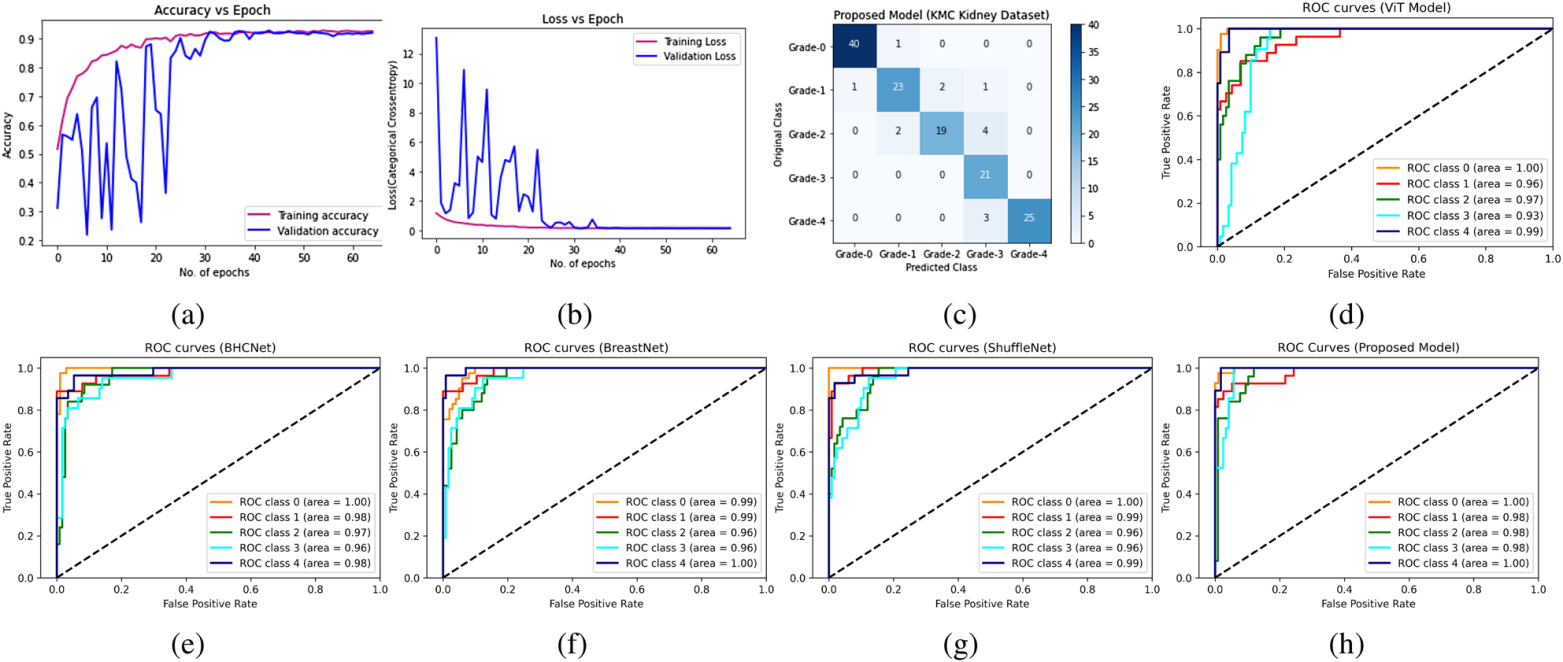
Learning curve of RCCGNet on intoduced KMC kidney dataset. (**a**) Training and validation accuracy of proposed model. (**b**) Training and validation loss of proposed model. (**c**) Confusion matrix of proposed model. (**d**–**h**) Receiver operating characteristic (ROC) curve of five best-performing state-of-the-art models using one versus rest approach.

**Table 5:** Overall quality comparison of proposed model with other competitive model (BreakHis dataset-Eight Class).

Metrics	Transfer learning approach			End-to-end trained deep learning networks					
	ResNet50 (2016)	IncResV2 (2016)	NASNet (2018)	ShuffleNet (2018)	BHCNet (2019)	BreastNet (2020)	LiverNet (2021)	ViT (2021)	RCCGNet (proposed)
Precision	0.7309	0.7678	0.7147	0.7897	0.8671	0.7868	0.8361	0.7415	0.9062
Recall	0.6726	0.7642	0.7362	0.7507	0.8446	0.7313	0.7979	0.7083	0.8842
F1 Score	0.6954	0.7549	0.7026	0.7579	0.8504	0.7492	0.8156	0.7170	0.8890
Accuracy	0.7384	0.7879	0.7755	0.8018	0.8823	0.7988	0.8529	0.7956	0.9009

To avoid any biased advantage we used a diverse set of quality metric which is *precision*, *recall*, *F1 score*, and *accuracy* which includes class-wise and overall score of the model. Overall accuracy is not the average of class-wise accuracy, it is model accuracy based on total TP, TN, FP, and FN instances belonging to all classes. These values are calculated using scikit-learn library in python and it is possible that the overall metric is lower than the lowest metric of an individual class. For KMC kidney dataset the proposed RCCGNet achieve 90.14% classification *accuracy* which is best among any comparison model. *Precision*, *recall*, and *F1-score* values of the proposed RCCGNet are 89.78%, 89.60%, and 89.06% respectively. Resnet-50 and InceptionResNetV2 utilizes transfer learning technique shows poor performance among all comparision models. Figure 4 shows the learning curves of the proposed RCCGNet. In Fig.4a,b, training curves and validation curves are very close to each other. This indicates training data best represents the validation data. The model which has training and validation curves closer to each other is robust during testing. The performance of a classifier can also be described by the confusion matrix itself. Figure 4c is the classification report of the proposed model expressed in terms of the confusion matrix. Out of 142 instances in the test set proposed model predicts 128 cases correctly which is the maximum compared to any reference models. In Grade-0 and Grade-3 case there is minimum misclassified samples in the proposed model. Receiver operating characteristic (ROC) curve and area under the curve (AUC) indicates how much a classification model is capable to distinguish different grades. It is possible that some model which is not the best-performing model can perform better for a particular class. The average AUC of the proposed model is best compared to all reference models. The ROC-AUC curve of five best-performing state-of-the-art models using one versus rest approach is shown in Fig. 4d–h. To verify the validity of RCCGNet on proposed kidney KMC dataset, experiments conducted on a well established BreaKHis histopathology dataset having eight classes, including all the breast cancer subcategories. Table 5 shows performance of different models for classification of breast tissues from BreaKHis histopathology dataset. For BreaKHis dataset, the proposed RCCGNet achieve 90.09% classification *accuracy*. *Precision*, *recall*, and *F1-score* values of the proposed RCCGNet are 90.62%, 88.42%, and 88.90% respectively. On BreaKHis dataset also the proposed RCCGNet is best perfoming model, this suggests that the proposed model is generalized independent of the dataset.

## Discussion

**Figure 5:**
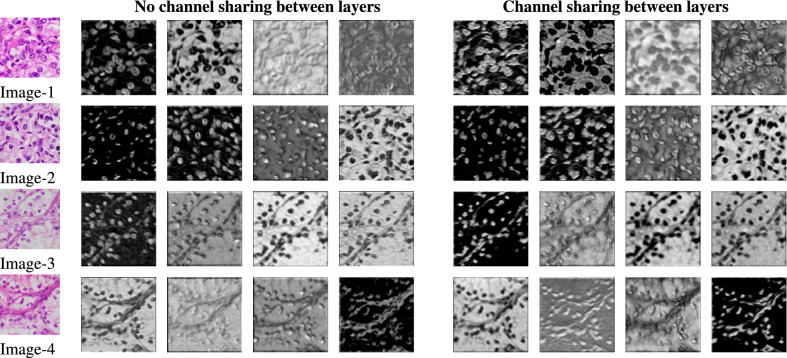
Comparison of intermediate features with and without channel sharing between layers.

**Figure 6:**
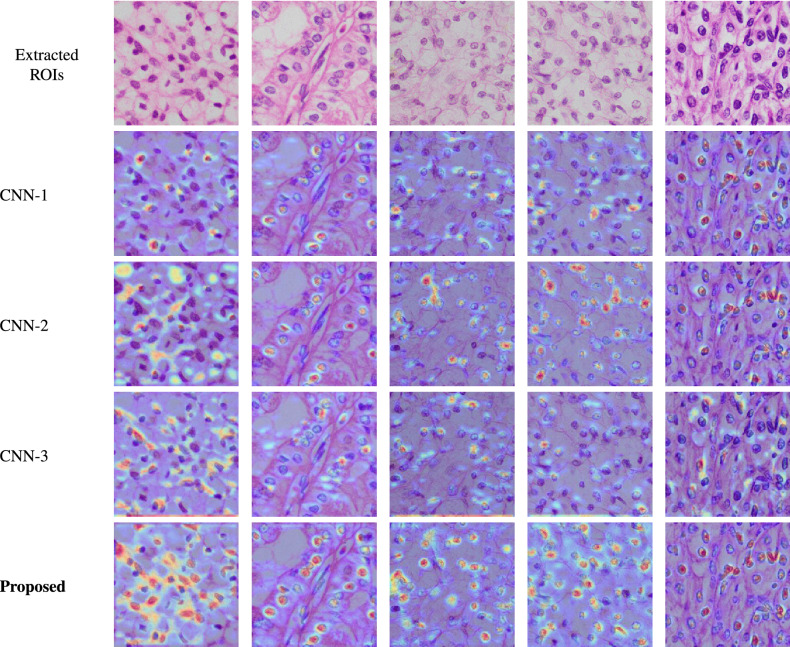
Visual comparison of different CNN variations using activation maps (red: very high probability score regions, orange: medium probability score regions, light blue: low probability score regions).

The core idea of RCCGNet lies in the proposed shared channel residual (SCR) block. The SCR block shares the channel with the next higher-order layer. The proposed model contains two paths of shared information with a simple gating mechanism, residual connections, convolutional, and fully connected layers. To measure the effectiveness of the main components of RCCGNet, these components are detached from the proposed model and made different variations of proposed model. These variations were trained in the same environment. Visual performance of each variation using intermediate features and activation map was compared with the proposed RCCGNet where all the important modules jointly worked. Comparison of intermediate features with and without channel sharing between layers for KMC kidney dataset is shown in Fig. 5 . Visual comparison of different CNN variations using activation map for KMC kidney dataset is shown in Fig. 6.

### SCR block without channel sharing

SCR block without channel sharing is a simple version of the proposed model. It has also two parallel paths and one skip connection. As discussed in Fig. 3, the first stage of the SCR block accepts an input that undergoes a convolution operation. In the simplified version both the input and feature map obtained after the convolution operation goes straight to the gating block without channel sharing. To verify the validity of the channel sharing method in the proposed SCR block, the extracted intermediate features of the proposed model, where the information is shared with the higher-order layer and a version of the proposed model which does not share the information between layers were analyzed and compared. The second stage of both CNN versions produces 32 feature maps, out of which 4 feature maps of both cases are analyzed. These four intermediate features are of four different grades. In most cases, nuclear boundaries are more clear and expressive when produced by sharing information between channels. The shape and size of nuclei are important parameters in grading cancerous tissue. The proposed method produces visible and sharp boundaries which help the model to learn the class representative features. The effect of channel sharing between layers can be observed in Fig. 5. The visuals of intermediate features also indicated that the proposed SCR block better segregates the object and background region. Without channel sharing, 2.3% drop in test accuracy were observed. Further, a drop in the value of F1 score, recall, and precision was also observed, which are 2.3%, 2.24%, and 3.06% respectively. From these observations, it is clear that in the proposed SCR block, inter-channel information sharing boosts the grading scores and effective utilization of this module in the proposed work was found through this ablation study.

### Effect of gating mechanism

Attention block helps the model to separate the most relevant nuclear region from background regions. The effect of removing the attention block can be seen in Fig. 6 of RCCGNet variation CNN-2. It also improves the network performance as the proposed RCCGNet produced higher values for both the datasets. Thus, the contribution of the attention block can not be ignored and it’s imperative to include it in the proposed new block.

### Comparision of activation maps of different variations

The proposed model is composed of several blocks. This part demonstrates the visual performance of the aforementioned blocks using activation maps produced with the help of gradient-weighted class activation mapping (Grad-CAM). The classification model in deep learning assigns a probability score to the final stage of the classifier based on extracted critical features. An activation map is a simple and straight-forward visualization technique to show the assigned probability to the relevant regions. How well each variation of the proposed model segregate the tissue regions from background pixels can be visualized by using activation maps shown in Fig. 6. Activation maps produced in CNN-1, where the model is not sharing inter-channel information, high probability regions are less, and some critical nuclear regions are missed to assign any probability. In CNN-2, where the gating mechanism is absent, it assigns some probability scores to irrelevant regions for some grades, which is not desirable. False-positive cases are maximum in CNN-2 compared to any other variations. Total number of detected nuclei are more in CNN-2 compared to CNN-1. In CNN-3, the complete SCR block is absent. The effect of removing the proposed SCR block can be observed in CNN-3 variation. Only few tissue regions get identified in the CNN-3 variation and the total detected nuclei are less compared to any other variations. Many relevant nuclear regions are assigned by lesser probability scores which results the number of partially detected nuclei being more in CNN-3. Whereas the proposed RCCGNet, where all the important components jointly worked, segregates tissue regions from background pixels better than compared to all other versions. The number of not detected and partially detected nuclei are very less in number in the proposed RCCGNet. In the RCCGNet, most of the tissue regions are clearly identified with high probability scores for all grades of images.

Figures 5 and 6 shows that the proposed RCCGNet is the best performing combination among all modifications made in the base model. This ablation study helps us to choose the best performing combination among all variations. The three important components togetherly making the proposed RCCGNet very effective in feature extraction with minimum computational complexity.

### 3-fold cross validation

To avoid any possible overfitting issue the proposed model and all reference models are tested using 3-fold cross-validation. Three folds of each class images were made to accomplish this. Models are trained for the first run using fold 2 and fold 3 and tested using fold 1. Model are trained in the second run using folds 1 and fold 3 and tested in fold 2. Similarly, for third run the models are trained using fold 1 and fold 2 and tested using fold 3. Table 6 lists the average outcomes of all three runs for the proposed model and the benchmark models. The suggested model produces nearly identical results to those shown in Table 4. ResNet^12^ and ShuffleNet^24^ show considerable variations in the result of Tables 4 and 6.

**Table 6:** 3-Fold cross validation average quality metrics comparison of proposed RCCGNet with other competitive models (KMC kidney dataset).

Metrics	Transfer learning approach				End-to-end trained deep learning networks					
	Grade	ResNet50 (2016)	IncResV2 (2016)	NASNet (2018)	ShuffleNet (2018)	BHCNet (2019)	BreastNet (2020)	LiverNet (2021)	ViT (2021)	RCCGNet (proposed)
Precision	0	0.7917	0.8754	0.8891	0.8360	0.8963	0.9522	0.9345	0.8974	0.9390
	1	0.6759	0.7307	0.6851	0.6919	0.8017	0.8241	0.8511	0.7606	0.9320
	2	0.6795	0.6635	0.7829	0.7921	0.8840	0.7792	0.8305	0.8185	0.9361
	3	0.6755	0.6215	0.7057	0.6499	0.8414	0.7588	0.8033	0.6980	0.8933
	4	0.7369	0.7955	0.8904	0.7668	0.9390	0.8167	0.8765	0.8233	0.9475
	Overall	0.7119	0.7373	0.7906	0.7473	0.8725	0.8262	0.8592	0.7996	0.9296
Recall	0	0.8475	0.8124	0.8621	0.8816	0.9803	0.8913	0.9018	0.9014	0.9607
	1	0.6814	0.7555	0.7925	0.6962	0.8592	0.7925	0.8148	0.7703	0.8888
	2	0.6920	0.7265	0.6968 ara>	0.7333	0.7884	0.8031	0.9115	0.7904	0.9357
	3	0.4583	0.575	0.6833	0.55	0.8	0.7333	0.7916	0.7083	0.9416
	4	0.7930	0.7064	0.8711	0.8432	0.8644	0.9007	0.8646	0.8146	0.8931
	Overall	0.6944	0.7152	0.7812	0.7408	0.8585	0.8242	0.8569	0.7970	0.9240
F1 Score	0	0.8127	0.8414	0.8746	0.8569	0.9365	0.9203	0.9152	0.8993	0.9490
	1	0.6710	0.7355	0.7253	0.6935	0.8286	0.8053	0.8301	0.7645	0.9035
	2	0.6792	0.6537	0.7306	0.7612	0.8283	0.7858	0.8668	0.8037	0.9327
	3	0.5249	0.5710	0.6881	0.5952	0.8193	0.7430	0.7881	0.7016	0.9148
	4	0.7631	0.7428	0.8805	0.8002	0.9001	0.8491	0.8702	0.8171	0.9190
	Overall	0.6902	0.7089	0.7798	0.7415	0.8625	0.8207	0.8541	0.7972	0.9238
Accuracy	0	0.8877	0.9153	0.9308	0.9168	0.9626	0.9569	0.9544	0.9432	0.9709
	1	0.8754	0.8989	0.8824	0.8850	0.9335	0.9292	0.9376	0.9113	0.9653
	2	0.8892	0.8639	0.9184	0.9209	0.9445	0.9263	0.9513	0.9334	0.9763
	3	0.8725	0.8696	0.8988	0.8753	0.9418	0.9156	0.9294	0.9002	0.9709
	4	0.9044	0.9057	0.9542	0.9197	0.9626	0.9390	0.9502	0.9293	0.9695
	Overall	0.7146	0.7268	0.7924	0.7589	0.8726	0.8336	0.8615	0.8088	0.9265

**Figure 7:**
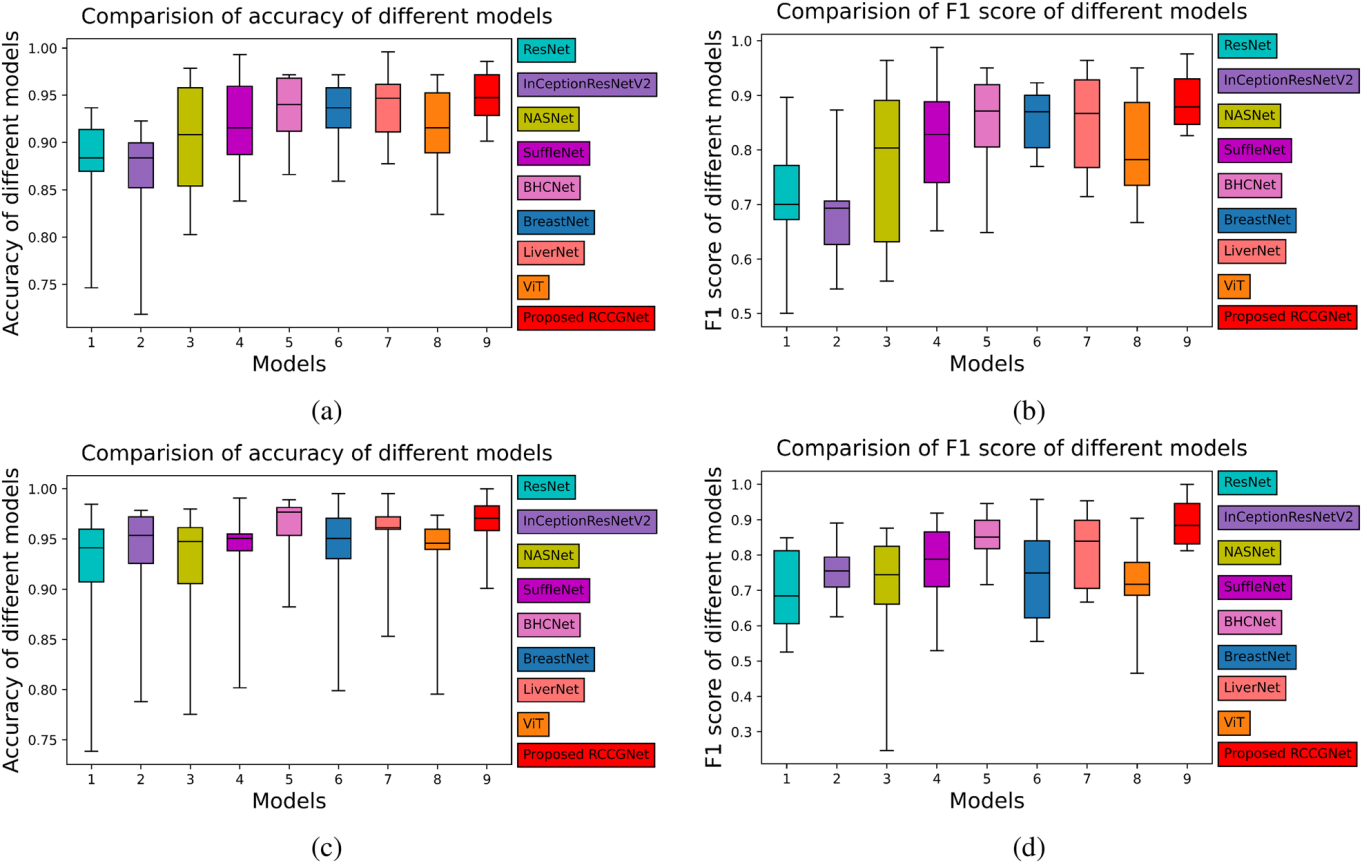
Box plot: (**a**,**b**) accuracy, F1 Score of different models for proposed KMC kidney dataset. (**c**,**d**) Accuracy, F1 Score of different models for BreakHis dataset.

### Statistical analysis

Depicting data using box plot is a standard method where it distribute the whole data points between five regions. It is a very good measure to check the dispersion of data between minimum to maximum via first quartile, median, and second quartile. *Accuracy*, *F1 score* values of different models on both datasets is presented using box plot in Fig. 7. Each of the boxes in the box plot contains a grade-wise score and overall score of a model. Performance variability of the grade-wise and overall score depends on the statistical distribution of values obtained by all classification models for both datasets. Box plot shows that metrics, *accuracy* and *F1 score* of RCCGNet, the median value, first quartile value, and third quartile value are higher compared to the reference models.

### Computational complexity analysis

The computational complexity of all the classification models is expressed in terms of total trainable parameters and floating-point operations (FLOPs). These values for proposed RCCGNet and all reference models are shown in Table 7. The proposed RCCGNet uses 0.3651 million parameters which is the least among all reference models except BHCNet. BHCNet has marginal difference in trainable parameter compared to proposed RCCGNet and it uses 0.3034 million parameters. NASNet uses least number of FLOPs. InceptionResNetv2 uses highest number of trainable parameters and FLOPs.

**Table 7:** Computational complexity comparison of architectures.

Model	Parameters (M = $10^6$)	FLOPs (G = $10^9$)
ResNet-50 (2015)	23.60 M	7.75 G
InceptionResNetV2 (2016)	54.34 M	13 G
NASNet (2017)	4.275 M	1.15 G
ShuffleNet (2018)	23.83 M	6.69 G
BHCNet (2019)	0.3034 M	4.46 G
BreastNet (2020)	0.6064 M	5.68 G
LiverNet (2021)	0.5740 M	3.72 G
ViT (2021)	45.26 M	33.03 G
Proposed RCCGNet	0.3651 M	4.48 G

### Conclusion

This work designed a fully automated deep learning framework called a Renal Cell Carcinoma Grading Network (RCCGNet) for the detection of malignancy levels of renal cell carcinoma (RCC) in kidney histopathology images. This paper is the first to propose an end-to-end automatic grading of kidney cancer from kidney histopathology images that were not yet focused on. In addition, we also introduced a novel kidney cancer dataset validated by skilled medical experts. Extraction of a class-specific representative set of features was possible due to the effective utilization of inter-channel information exchange at three different resolutions within the network. Residual connection, Information sharing between different layers, and gating mechanism in shared channel residual network were attributed to the distinguishing performance. We explicitly performed data augmentation techniques to handle the class imbalance problem. The performance of the proposed RCCGNet was evaluated by the most preferred quality metrics and achieved 90.14% of *accuracy*, and 89.06% *F1-score* on the proposed KMC kidney dataset, and on the BreakHis dataset, the proposed RCCGNet achieved 90.09% of *accuracy*, and 88.90% *F1-score*. Experimental results show that the proposed RCCGNet has the potential to grade five different grades associated with kidney cancer histopathology images with better accuracy. Our approach is generalized and effectively works on multiple organ histopathology datasets. It reduces the need for extensive computational complexity. In the future, we will focus on the considerable extension of the kidney dataset and other pathological datasets.

## Data Availability

The datasets used and/or analyzed during the current study are available from the corresponding author on reasonable request.
